# Thalamic functional connectivity and its association with behavioral performance in older age

**DOI:** 10.1002/brb3.943

**Published:** 2018-02-27

**Authors:** Aimée Goldstone, Stephen D. Mayhew, Joanne R. Hale, Rebecca S. Wilson, Andrew P. Bagshaw

**Affiliations:** ^1^ Centre for Human Brain Health University of Birmingham Birmingham UK; ^2^ School of Psychology University of Birmingham Birmingham UK

**Keywords:** aging, memory, reaction time, resting‐state, thalamo‐cortical, thalamo‐hippocampal

## Abstract

**Introduction:**

Despite the thalamus’ dense connectivity with both cortical and subcortical structures, few studies have specifically investigated how thalamic connectivity changes with age and how such changes are associated with behavior. This study investigated the effect of age on thalamo‐cortical and thalamo‐hippocampal functional connectivity (FC) and the association between thalamic FC and visual–spatial memory and reaction time (RT) performance in older adults.

**Methods:**

Resting‐state functional magnetic resonance images were obtained from younger (*n* = 20) and older (*n* = 20) adults. A seed‐based approach was used to assess the FC between the thalamus and (1) sensory resting‐state networks; (2) the hippocampus. Participants also completed visual–spatial memory and RT tasks, from the Cambridge Neuropsychological Test Automated Battery (CANTAB).

**Results:**

Older adults exhibited a loss of specificity in the FC between sensory thalamic subregions and corresponding sensory cortex. Greater thalamo‐motor FC in older adults was associated with faster RTs. Furthermore, older adults exhibited greater thalamo‐hippocampal FC compared to younger adults, which was greatest for those with the poorest visual–spatial memory performance.

**Conclusion:**

Although older adults exhibited poorer visual–spatial memory and slower reaction times compared to younger adults, “good” and “poorer” older performers exhibited different patterns of thalamo‐cortical and thalamo‐hippocampal FC. These results highlight the potential role of thalamic connectivity in supporting reaction times and memory in aging. Furthermore, these results highlight the importance of including the thalamus in studies of aging to fully understand how brain changes with age may be associated with behavior.

## 1 INTRODUCTION

Several lines of evidence support the view that the thalamus, rather than acting primarily to integrate incoming sensory information and project it to the relevant cortical regions (Jones, 1985), has a much wider role and exerts a strong influence over cortical activity (Baxter, 2013; McAlonan, Cavanaugh, & Wurtz, 2008; O'Connor, Fukui, Pinsk, & Kastner, 2002; Purushothaman, Marion, Li, & Casagrande, 2012; Saalmann, 2014; Saalmann, Pinsk, Wang, Li, & Kastner, 2012; Theyel, Llano, & Sherman, 2010). The majority of thalamic inputs originate from the cortex, rather than sensory peripherals (Sherman & Guillery, 2013), and higher order thalamic nuclei receive dense input from cortical layers five and six, resulting in cortico‐thalamo‐cortical pathways which create indirect connections between cortical areas (Saalmann, 2014; Sherman & Guillery, 2013). Furthermore, every dorsal thalamic nucleus receives fibers back from the cortical region that it projects to, resulting in large‐scale cortical reciprocal connectivity (Jones, 1985; Sherman & Guillery, 2013). Although the precise function of this diffuse thalamo‐cortical and cortico‐thalamic connectivity remains poorly understood, it has been argued that higher order nuclei may function to modulate neuronal synchrony between different cortical regions and networks, to increase the efficiency of information transfer (see Saalmann 2014 for a review).

These recent studies provide convincing evidence for the role of the thalamus in modulating cortical activity, but how this thalamo‐cortical circuitry might be affected by advancing age, and whether changes to the thalamo‐cortical system relate to cognitive declines with age, has received surprisingly little attention. Declining memory performance is perhaps the cognitive deficit most commonly associated with advancing age (see Craik & Rose, 2012 and Khan, Martin‐Montanez, Navarro‐Lobato, & Muly, 2014 for reviews). While the hippocampus clearly plays a vital role in explicit (Riedel et al., 1999; Schacter, Alpert, Savage, Rauch, & Albert, 1996; Squire, 1992) and implicit (Duss et al., 2014) memory, the connectivity between the hippocampus and other brain regions, including the anterior thalamus, has also been implicated in supporting memory function (Aggleton & Brown, 1999; Aggleton et al., 2010; Child & Benarroch, 2013; Jankowski et al., 2013). Evidence from patients with thalamic infarcts supports the view that disrupted thalamo‐cortical structural connectivity is associated with memory problems (Serra et al., 2014), while functional connectivity (FC) strength between the dorsomedial nucleus of the thalamus and parts of the striatum has also been negatively associated with episodic memory functioning in 49‐ to 80‐year‐olds (Ystad, Eichele, Lundervold, & Lundervold, 2010). Processing speed, which commonly slows with age (Albinet, Boucard, Bouquet, & Audiffren, 2012; Der & Deary, 2006; Nilsson, Thomas, O'Brien, & Gallagher, 2014; Papp et al., 2014; Salthouse, 2009; Sliwinski & Buschke, 1999), has also been linked with increased thalamic fiber integrity in both young (Tuch et al., 2005) and older (Ystad et al., 2011) adults. Studies that have used measures of structural connectivity have suggested that the integrity of thalamic nuclei and their projections to cortical regions decline with age (Hasan et al., 2011; Hughes et al., 2012; Ota et al., 2007) and that these changes have implications for attention, processing speed, working, and episodic memory (see Fama and Sullivan 2015 for a review). Grieve, Williams, Paul, Clark, and Gordon (2007) reported decreased fractional anisotropy in frontal and parietal lobes and anterior thalamus with age which was associated with reduced executive function in older adults. Similarly, a DTI study of 121 participants (aged 18–61 year) observed that motor task performance was negatively associated with thalamo‐precentral gyrus connectivity (radial diffusivity), while better verbal memory scores were positively associated with the number of thalamic voxels characterized as being “connected” to frontal, parietal, and temporal ROIs (Philp, Korgaonkar, & Grieve, 2014). Furthermore, gross morphometric alterations to the thalamus with advanced age have also been reported (Goodro, Sameti, Patenaude, & Fein, 2012; Long et al., 2012; Serbruyns et al., 2015; Sullivan, Rosenbloom, Serventi, & Pfefferbaum, 2004), although not universally (Good et al., 2001).

Taken together, this previous research provides strong evidence for the thalamus’ role in cognition as well as its potential importance in mediating cognitive decline with age, via disrupted connectivity and changes to the structural and functional integrity of brain networks. However, the majority of studies in humans have investigated the thalamus as a whole, despite the differential connectivity and function of its subregions. Segmentation of the thalamus has been performed with diffusion tensor imaging (DTI) data (Behrens et al., 2003; Duan, Heckenberg, Xi, & Hao, 2006; Jang & Yeo, 2014; Kumar, Mang, & Grodd, 2014; Ye, Bogovic, Ying, & Prince, 2013) as well as using FC of resting‐state fMRI data (Hale et al., 2015; Kim, Park, & Park, 2013; Zhang et al., 2008). Despite some discrepancies in thalamic connectivity results between these different methodologies, the general principles identified are similar and reasonably consistent with histological and anatomical studies. Applying a parcellation of the thalamus allows for a more fine‐grained exploration of its heterogeneous structure and is necessary for a detailed understanding of thalamic function and its changes with aging. Here, we compare thalamo‐cortical FC between younger and older adults to investigate the association between thalamic connectivity and disruption of memory and processing speed. This is, to our knowledge, the first study to investigate the FC of thalamic subregions in older age. We focussed on the relationship between first‐order nuclei and sensory cortices, as these connections are better characterized and perhaps more intuitive to understand in terms of FC than the connectivity between higher order nuclei and higher cortical regions. We also investigated hippocampal–thalamic FC, across all subregions, given the importance of these connections to memory.

## 2 METHODS

### 2.1 Participants

Twenty younger (*M* = 27, ± 3 years, 10 male) and twenty older (*M* = 74, ± 4 years, nine male) participants took part. Older participants were screened for cognitive impairment with the Advanced Mini‐Mental State Test (3MS) (Teng & Chui, 1987); the group's average score was 97.65 (±2.6, range: 88–100). No participants scored below the cutoff (79/100) for normal cognitive ability. All participants (excluding two younger participants for whom English was not their native language) also took part in the National Adult Reading Test (NART) as an estimator of IQ (Nelson & Willison, 1991). Younger participants had an average “full IQ” score of 114 (±7.56, range: 97–124), compared to a score of 119 (±7.07, range: 106–131) for the older participants. IQ scores were not significantly different for the two groups, as assessed by a one‐way anova (*F*(1,37) = 3.811, *p* = .059).

### 2.2 Procedure

Participants gave written informed consent, and the study was approved by the Research Ethics Board of the University of Birmingham. All participants were screened for MR compliance and completed the NART and 3MS. Participants then underwent the MRI session. During the resting‐state scan, participants were asked to keep their eyes open and think of nothing in particular. Approximately twenty minutes after the MRI session, participants completed tests of memory and reaction time (simple reaction time: SRT), from the Cambridge Neuropsychological Test Automated Battery (CANTAB, Cambridge Cognition). All tasks were computed in a quiet testing room, on an 11” Samsung tablet (XE700T1C; Intel 1.7 GHz i5 processor, 4GB RAM, 64‐bit Windows 7). Upon completion, participants were thanked and debriefed.

### 2.3 MRI procedure

A Philips Achieva 3T MRI scanner with a 32‐channel head coil was used to acquire MRI data. A fifteen‐minute resting‐state scan was acquired (T2*‐weighted fMRI data with whole brain coverage: 3 × 3 × 4 mm voxels, TR = 2,000 ms, TE = 35 ms, SENSE factor = 2, flip angle = 80°, and volumes = 450). In addition, a high‐resolution (1 mm isotropic) T_1_‐weighted anatomical image was obtained (TR = 8.4 ms, TE = 3.8 ms, flip angle = 8, matrix = 288 × 288, slice dimensions = 1 mm^3^, and 175 slices). During the resting‐state scan, participant's cardiac and respiratory cycles were measured using pneumatic bellows and a pulse oximeter. Foam padding was positioned around the head to reduce motion artifacts.

### 2.4 Neuroimaging methods

#### 2.4.1 Definition of sensory‐network ROIs

**Figure 1:**
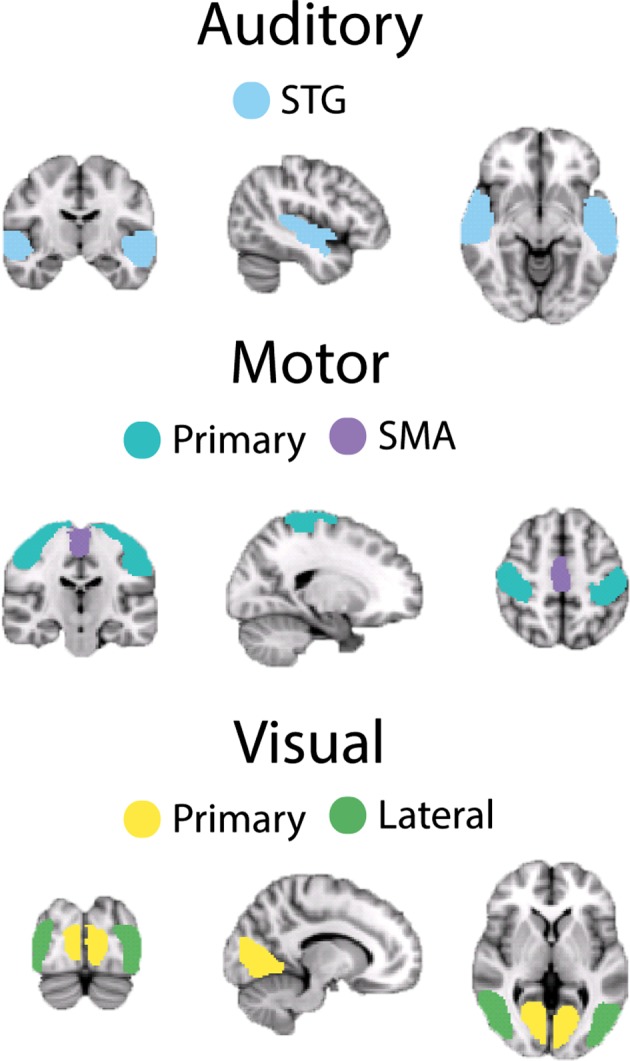
Illustration of each node of the three main sensory RSNs: auditory, motor and visual. Auditory comprises *STG* superior‐temporal gyrus. Motor comprises *M1* primary motor cortex, *SMA* supplementary motor area. Visual comprises primary and lateral visual cortices

The spatial location of each resting‐state network's (RSN) individual nodes was defined from six‐minute resting‐state scans (3 × 3 × 4 mm voxels, TR = 2,000 ms, TE = 35 ms, flip angle 80°, and SENSE factor = 2) acquired from an independent cohort of fifty‐five subjects (28 male, age 25 ± 4 years) which was collected as part of a previous study (Przezdzik, Bagshaw, & Mayhew, 2013). Using FSL 4.1.8 (http://www.fmrib.ox.ac.uk/fsl), data were motion corrected, spatially smoothed (5 mm), temporally concatenated across subjects, and decomposed into 20 spatially independent components using MELODIC (Beckmann and Smith, 2004). Visual, auditory, and motor networks were visually identified from individual components, based on their spatial similarity to previous reports (Damoiseaux et al., 2006). Each component was thresholded at a *Z*‐statistic >4, based on previous methodology (Khalsa, Mayhew, Chechlacz, Bagary, & Bagshaw, 2013), to ensure that each of the network nodes was spatially distinct. Each network was then manually separated into its individual nodes (see Figure 1).

For each participant in this study, FLIRT (Jenkinson, Bannister, Brady, & Smith, 2002) was used to transform these node masks, which were created from an independent cohort, into native functional space, using the T_1_‐weighted image as an intermediate step. All subsequent analysis then took place in native (or individual) space. ROIs were defined as 5 × 5x5 voxel cubes centered on the maximum *Z*‐statistic voxel for each node (see Appendix: Table A1). In order to account for differences in the proportion of gray/white‐matter voxels within each ROI between the two age‐groups, FAST (Zhang, Brady, & Smith, 2001) was used to segment each individual's T_1_‐weighted image into gray matter, white matter, and CSF. These partial volume maps were then transformed into functional space using FLIRT (Jenkinson & Smith, 2001; Jenkinson et al., 2002), with nearest neighbor interpolation and a threshold of 0.5 to preserve approximately the size of the original partial volume map. Using this map, only gray‐matter voxels within each cortical ROI were included for functional connectivity (FC) analysis. Older adults were found to have significantly fewer remaining voxels within RSN node ROIs, as indicated by a significant main effect of age (*F*(1,38) = 47.23, *p* < .001, ɳ^2 ^= 0.554), but no significant age*node interaction (*F*(8, 304) = 1.07, *p* = .38, ɳ^2^ = 0.027). Appendix: Table A1 displays the average ROI sizes for the two age‐groups, after including only gray‐matter voxels.

#### 2.4.2 Definition of hippocampus and thalamus

In addition to the RSN ROIs defined from ICA, we also anatomically defined left and right hippocampal and thalamic nodes. This was done by thresholding the hippocampal and thalamic probability maps, provided by the Harvard–Oxford subcortical structural atlas included in FSL, to retain the top 75% when ordered in terms of probability values, in order to obtain reliable maps of these structures. These thresholded masks were then binarized and, as described above for the cortical ROIs, transformed into functional space for each participant. For the hippocampal nodes, the same method was applied to include only gray‐matter voxels for FC analysis.

#### 2.4.3 Segmentation of thalamus

**Figure 2:**
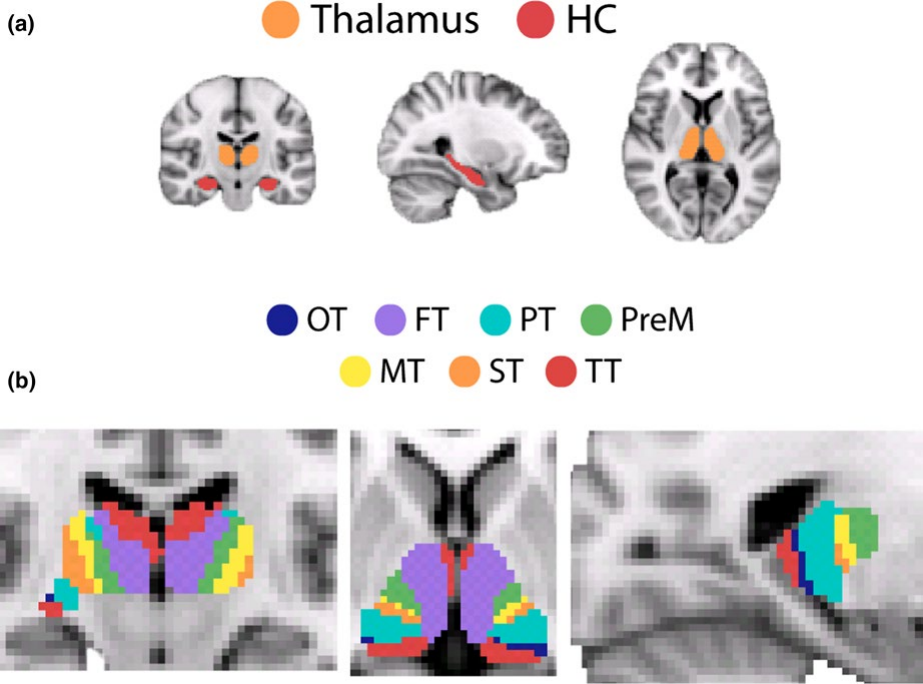
(a) Illustration of the anatomically defined masks for HC hippocampal complex and thalamus. (b) Depiction of each thalamic sub‐region from the Oxford Thalamic Connectivity Atlas (Behrens et al., 2003). The descriptions below detail the cortical region that each thalamic‐sub‐region is thought to be most strongly connected to and the corresponding thalamic nuclei each sub‐region is said to contain. *OT* visual cortex (LGN, inferior pulvinar and some intralaminar nuclei), *FT* frontal cortex (some of MD, VA, parts of anterior complex), *PT* posterior parietal cortex (anterior pulvinar), *pMT* pre‐motor cortex (VLa and VA), *MT* primary motor cortex (VLp), *ST* somatosensory cortex (LP and VPL), *TT* temporal cortex (some of MD, parts of anterior complex, medial and inferior pulvinar)

In order to conduct more fine‐grained FC analysis of the thalamus, we used the Oxford thalamic connectivity atlas (http://fsl.fmrib.ox.ac.uk/fsl/fslwiki/Atlases; Behrens et al. (2003)). The probabilistic masks from this atlas, thresholded at a probability of 25% as applied previously (Serra et al., 2014), comprise seven bilateral subregions that have been identified to structurally connect predominantly to the following: primary motor cortex (MT), premotor cortex (pMT), somatosensory cortex (ST), occipital cortex (OT), frontal cortex (FT), posterior parietal cortex (PT), and temporal cortex (TT) (see Figure 2). Each of these thalamic subregions was transformed into functional space for each participant (as described above in Section 2.4.1). As gray‐matter segmentation of the thalamus was not adequate for all participants, we chose instead to exclude any voxels that had been identified as CSF (using the partial volume maps described in Section 2.4.1) to control for potential differences in thalamic morphometry between the two age‐groups. Older adults were found to have significantly fewer remaining voxels within thalamic subregion ROIs, as indicated by a significant main effect of age (*F*(1,38) = 17.95, *p* < .001, ɳ^2^ = 0.321). A significant age*subregion interaction (*F*(1.42, 53.8) = 13.37, *p* < .001, ɳ^2^ = 0.26) revealed that this was the case for all subregions (*p* < .005), excluding pMT (*p* = .18) and MT (*p* = .11). Appendix: Table A2 displays average thalamic and hippocampal ROI sizes for the two age‐groups.

### 2.5 Functional connectivity analysis

The effect of respiratory and cardiac confounds (corrected using RETROICOR) (Glover, Li, & Ress, 2000) and subsequently variations in breathing depth and heart rate interval (Birn, Diamond, Smith, & Bandettini, 2006; Chang, Cunningham, & Glover, 2009) were reduced using custom MATLAB code. Data were then preprocessed according to standard methodology prior to FC analysis (Fox et al., 2005). Data were motion and slice‐time corrected, spatially smoothed (5 mm), and high‐pass filtered using FEAT. Data were then temporally band‐pass filtered (0.009 < Hz<0.08), and further potential confound signals were removed using multiple linear regression: the six motion parameters of head rotation and translation, white matter and CSF signals, and the global signal, calculated by averaging the BOLD time series across all brain voxels. FC strength was then calculated as the correlation coefficient (Pearson's *r*) between the mean BOLD time series from thalamic subregion and the mean BOLD time series of each RSN node. Correlation coefficients were converted to a normal distribution using Fisher's *r*‐to‐*z* Transform (*z* = 0.5 Ln [(1 + *r*) / (1 − *r*)]) (Jenkins & Watts, 1968). These values were converted into *z*‐scores by dividing by the square root of the variance (1/√ (*n*−3), where *n* is the degrees of freedom in the measurement (i.e., number of volumes‐2).

#### 2.5.1 Thalamic subregions to sensory RSNs

We explored the differences between thalamic‐sensory FC for the two age‐groups by assessing FC between sensory thalamic subregions and sensory RSNs. FC was calculated by seeding from each of the primary sensory (primary motor, occipital) and the temporal thalamic subregions to each of the nodes of the sensory RSNs.

#### 2.5.2 Thalamic subregions to hippocampus

Age‐related thalamo‐hippocampal FC differences were also investigated by calculating FC between each of the thalamic subregions and left and right hippocampi.

### 2.6 Behavioral measures

Paired associates learning (PAL) task and simple reaction time (SRT) task from the CANTAB battery were employed as measures of memory (visual–spatial) and reaction time, both of which are affected by advancing age (Der & Deary, 2006; Dykiert, Der, Starr, & Deary, 2012; Petersen, Smith, Kokmen, Ivnik, & Tangalos, 1992; Skolimowska, Wesierska, Lewandowska, Szymaszek, & Szelag, 2011; Sliwinski & Buschke, 1999). Computer expertise was not required to complete these tasks as responses were recorded via a touch screen (PAL) and a button box (SRT). This ensured that any differences in computer familiarity between the two age‐groups did not confound performance.

#### 2.6.1 Paired associates learning

This task is a measure of visual–spatial memory. Patterns are displayed to participants one at a time in a number of locations on the screen. After all patterns have been displayed, participants must select the location of each pattern when prompted.

The outcome measure used to assess memory performance was the number of errors made at stage 7 of the task (where six patterns are displayed); thus, a lower score indicates better performance. This measure was selected as it is sensitive to memory impairment (Sahakian & Owen, 1992) and capable of distinguishing patients with Alzheimer's disease from healthy older controls with an accuracy of 98% (Swainson et al., 2001).

#### 2.6.2 Simple reaction time

SRT delivers a known stimulus to a known location to elicit a known response. In this task, a stimulus (a white square) was presented on a black background and participants were instructed to respond by pressing a key on a two‐button button box whenever they saw the stimulus appears on the screen. The only uncertainty is when the stimulus will appear, as there is a variable interval between the previous trial response and the onset of the stimulus for the next trial. An initial practice block of 24 trials familiarized participants with the task. Following this, participants completed two assessment blocks of 50 trials each. As older age is associated with slowing of reaction times (Der & Deary, 2006; Dykiert et al., 2012; Woods, Wyma, Yund, Herron, & Reed, 2015), the outcome measure used to assess performance on this task was mean reaction time (RT).

### 2.7 Statistical analysis

#### 2.7.1 Good vs. poor performers

To address the question of whether any age‐related differences in FC are beneficial or detrimental, we assessed whether older “good” performers had thalamic FC that was more similar to younger participants than “poor” performers in their own age‐group. For this, older adults were split into good and poor performers based on a median split of the group's memory or reaction time performance. This meant that, for PAL, participants with <6 errors on stage 7 of the task were classified as “good performers,” while those with >6 were classed as “poor performers.” FC of good and poor performers was then compared to younger performers (two of whom were excluded from this analysis for having errors >6, which was the criterion for an older, “poor” performer on this task). Final sample sizes using this categorization were as follows: 18 younger, nine older good, and 11 older poor performers. Splitting the participants in this way resulted in similar categorizations to the normative data available from CANTAB. All of the participants we categorized as “good performers” fell within the normalized “healthy” range (normalized z‐scores ranging from 0.60 to 1.2), of our “poorer performers,” eight of 11 had “impaired” scores (−2.12 to −0.08), and the three that were not categorized as impaired were very close to the “impaired” threshold (<0), with scores of 0.02–0.08 which were all much less than our lowest score in the “good performer” group.

For SRT, participants with RTs shorter than the median value of 295 ms were classified as “good” performers, while those with RTs above 295 ms were classified as “poor”. FC of good and poor performers was then compared to younger performers (three of whom were excluded from this analysis for RTs >295 ms, which was the criterion for an older, “poor” performer on this task). Final sample sizes using this categorization were as follows: 17 younger, 10 older good, and 10 older poor performers. Normative data were not available from CANTAB for this task, so comparison of our good/poor groups to normalized z‐scores was not possible. For both tasks, no significant difference in age was found between good and poor participants, indicating that performance differences were not driven by chronological age alone and that “poor” performers were not simply the oldest participants in the group (see Table 1).

**Table 1:** Age details for the older participants when divided into “good” or “poor” performers for the two cognitive tasks (PAL, paired associates learning; SRT, simple reaction time). For both tasks, the two performance groups did not differ significantly in age, suggesting that performance differences were not driven by chronological age alone. Statistical outcomes from the t tests comparing the ages for the two groups are displayed for each task. Mean age, median age, and the range of ages are shown for each performance group for the two tasks

	PAL			SRT		
	*t*(18) = 0.72, *p* = .479			*t*(18) = 0.24, *p* = .815		
	Mean	Median	Range	Mean	Median	Range
Good	74.41	74.98	69–78	73.85	72.16	66–81
Poor	72.96	73.35	66–81	73.37	75.43	73–79

To link FC with task performance, we focussed on paired connections which we hypothesized would be most relevant to the two tasks. The hippocampus is a vital structure for memory formation and retrieval (for reviews see Bird & Burgess, 2008 & Squire, 1992) and, specifically, spatial memory (Burgess, Jeffrey, & O'Keefe, 1999; Cohen et al., 1999; Eichenbaum, Dudchenko, Wood, Shapiro, & Tanila, 1999). In addition, the role of the hippocampal–anterior thalamic axis is implicated in memory processes (Aggleton & Brown, 1999; Jankowski et al., 2013; Warburton, Baird, Morgan, Muir, & Aggleton, 2001). We therefore sought to investigate whether differences in thalamo‐hippocampal FC with age were associated with memory performance on the PAL task. For SRT performance, we examined thalamic‐motor FC.

#### 2.7.2 Correlations between FC and behavioral performance

In addition to the analyses described above, we correlated FC strength with PAL and SRT performance in order to assess whether there was a linear relationship between thalamic FC and behavioral performance. For PAL, we correlated thalamo‐hippocampal FC, for each thalamic nuclei. For SRT, we correlated thalamo‐motor cortex FC for each of the first‐order nuclei (for reasons described in 2.7.1). For each of the analyses, we chose to perform correlations separately for younger and older participants, due to the large differences in their performance on the two tasks.

#### 2.7.3 Specific analyses

IBM SPSS Statistics for Windows (version 20.0) was used for all statistical analyses. All pairwise comparisons were corrected for multiple comparisons with false discovery rate. Only *p*‐values with a FDR <5% are highlighted as significant, any *p*‐values reported from pairwise comparisons are adjusted. For anovas where the principle of sphericity was violated, Greenhouse–Geisser correction was applied to degrees of freedom. As the measures of SST and PAL violated assumptions of normality, they were log‐transformed before computing correlational analysis. For PAL, a constant of 1 was added to scores of 0 before transformation. For all correlation analyses with behavioral measures, age, gender, handedness, and NART score were included as covariates of no interest.

We assessed whether FC between thalamic subregions and RSNs differed with age using mixed design anovas with three factors as follows: age, RSN node and thalamic subregion, and their interaction terms. Finally, mixed design anovas with three factors, performance group (i.e., younger, old good performers, and old poor performers), RSN node and thalamic subregion, and their interaction terms, were used to assess whether older “good” performers had thalamic FC that was more similar to younger participants than “poor” performers in their own age‐group.

## 3 RESULTS

### 3.1 Behavioral results

**Figure 3:**
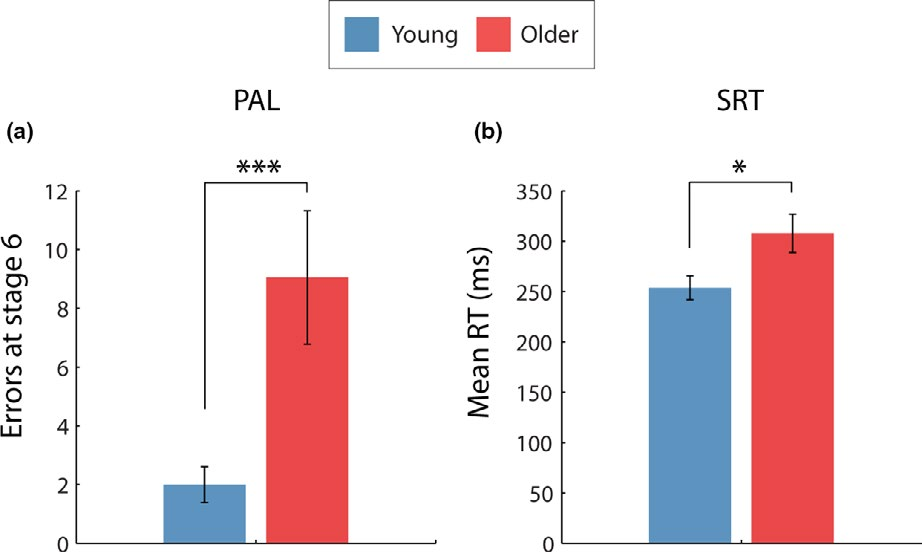
The average number of errors made on stage 7 (6 boxes all containing a pattern) of the PAL task for younger and older adults (a). The average reaction times on the SRT task for younger and older adults (b). ***p = .005, *p < .05. Error bars represent standard error, calculated across participants

Average performance on the two tasks is shown in Figure 3. ancovas were used to assess differences between the two age‐groups while controlling for NART performance (a measure of estimated IQ). Older adults made significantly more errors on stage seven of the PAL task (*F*(1, 37) = 17.76, *p* < .001) and had significantly slower RTs (*F*(1,37) = 5.69, *p* = .022) compared to younger participants, even after controlling for an estimate of IQ. For PAL, the number of errors ranged from 0 to 10 for younger adults and 0 to 38 for older adults. For SRT, RT ranged from 187.36 to 379.91 ms and 198.35 to 420.63 ms for older adults. As stated in section 2.7.1, two to three younger participants were excluded from further analysis exploring relationships between FC and cognition as they had RTs or PAL scores that met the threshold for the “older poor” group, RTs ranged from 187.36 to 279.91 ms and PAL errors ranged from 1 to 5 for the remaining younger participants.

### 3.2 Head motion

Younger and older adults did not differ significantly in terms of relative or absolute head motion parameters as revealed by a nonsignificant main effect of age (*F*(1,38) = 0.13, *p* = .73, ɳ^2^ = 0.003) and a nonsignificant age*motion interaction (*F*(1,38) = 0.46, *p* = .50, ɳ^2^ = 0.01). On average, younger adults had 1.43 ± 0.33 mm of absolute and 0.08 ± 0.04 mm relative head motion, compared to older adults who had 1.41 ± 0.36 mm and 0.13 ± 0.07 mm, respectively.

### 3.3 Thalamic FC

#### 3.3.1 Thalamo‐hippocampal FC

**Figure 4:**
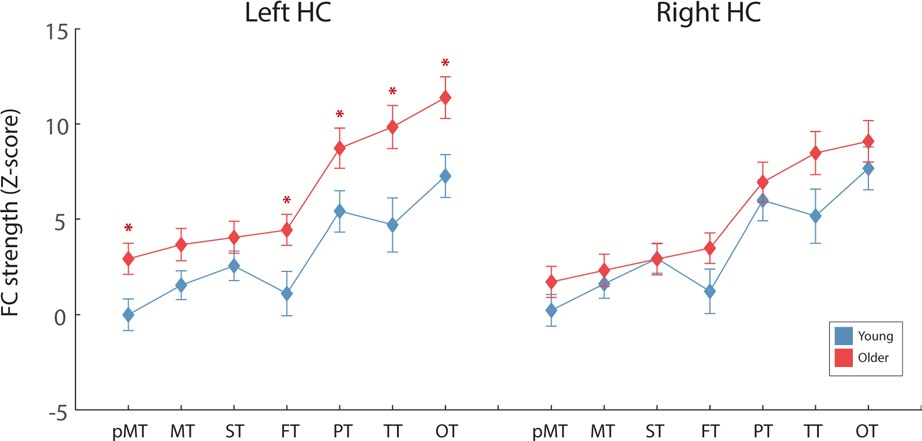
The average thalamo‐left hippocampaland thalamo‐right hippocampal FC for the two age groups, for each thalamic sub‐region. Across sub‐regions, older adults exhibited significantly greater thalamo‐left hippocampal FC, compared to younger. For thalamo‐right hippocampus, no significant difference in FC strength was identified between the two groups. Error bars represent standard error, calculated across participants. *p < .05 after FDR correction

Older adults exhibited significantly greater thalamo‐left hippocampal FC, averaged across all subregions of the thalamus, as indicated by a significant main effect of age (*F*(1,38) = 6.54, *p* = .015, ɳ^2^ = 0.147) and a nonsignificant age*subregion interaction (*F*(2.30, 73.64) = 3.0, *p* = .058, ɳ^2^ = 0.073) (see Figure 4a). However, younger and older adults did not differ in terms of thalamo‐right hippocampal FC, as revealed by a nonsignificant main effect of age (*F*(1,38) = 1.06, *p* = .310, ɳ^2 ^= 0.027) and a nonsignificant age*subregion interaction (*F*(2.03, 77.28) = 2.17, *p* = .120, ɳ^2^ = 0.054) (see Figure 4b). For both hippocampi, independent of age, FC was found to vary with thalamic subregions as indicated by a significant main effect of subregion.

#### 3.3.2 Thalamic‐sensory cortex FC

##### Auditory RSN

The two age‐groups did not differ in average thalamic‐auditory RSN FC, across thalamic subregions (*F*(2, 76) = 3.06, *p* = .06, ɳ^2^ = 0.08) or RSN nodes (*F*(1,38) = 0.26, *p* = .61, ɳ^2^ = 0.01). Similarly, there was no significant interaction between age*thalamic subregion*RSN node (*F*(1.6, 58.89) = 1.37, *p* = .26, ɳ^2^ = 0.04).

##### Motor RSN

Thalamic‐motor FC differed significantly between the two age‐groups, dependent on subregion, as indicated by a significant thalamic region*age‐group interaction (*F*(1.6, 60.89) = 8.54, *p* = .001, ɳ^2^ = 0.18). Pairwise comparisons revealed that older adults exhibited greater TT–motor cortex FC (*p* = .015, ɳ^2^ = 0.15) compared to younger adults. Similarly, older adults showed greater TT‐left M1 FC (*p* = .005, ɳ^2^ = 19), TT–right M1 FC (*p* = .01, ɳ^2^ = 0.16), and OT‐right M1 FC (*p* = .03, ɳ^2^ = 0.12) compared to younger adults. Thalamic region‐SMA FC did not differ for the two age‐groups (MT‐SMA: *p* = .377, ɳ^2^ = 0.02, OT‐SMA: *p* = .413, ɳ^2^ = 0.02, TT‐SMA: *p* = .171, ɳ^2^ = 0.05).

**Figure 5:**
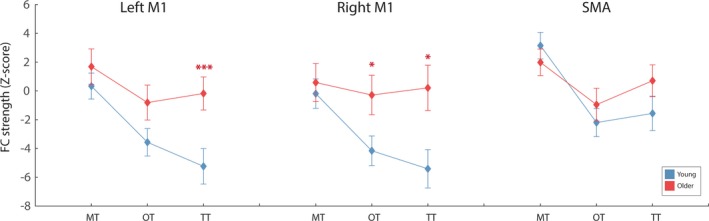
The average FC between each thalamic sub‐regions and each motor RSN node, for the two age groups. Older adults exhibited significantly greater OT – and TT‐ primary motor FC compared to younger adults. Error bars represent standard error, calculated across participants. *p < .05 and ***p < .005, after FDR correction

Thus, although older and younger adults did not differ in FC between MT and motor cortex, older adults had significantly greater TT–motor cortex FC compared to younger adults. This suggests that the FC specificity between MT and motor cortex, which is present in younger adults, was reduced for older adults. See Figure 5 for comparisons across thalamic subregions and age‐groups.

##### Visual RSN

The two age‐groups did not differ in average thalamic‐visual RSN FC, across thalamic subregions (*F*(2,76) = 0.25, *p* = .78, ɳ^2^ = 0.006) or RSN nodes (*F*(1.89, 71.67) = 2.30, *p* = .110, ɳ^2^ = 0.06). Similarly, there was no significant interaction between age*thalamic subregion*RSN node (*F*(3.63, 137.86) = 0.44, *p* = .76, ɳ^2^ = 0.01).

### 3.4 Thalamic FC and behavioral performance

#### 3.4.1 Thalamo‐hippocampal FC and memory performance

Older poor PAL performers had significantly greater thalamo‐right hippocampal FC, independent of thalamic subregion, compared to older good PAL performers (*p* = .023) and younger adults (*p* = .023), as revealed by a significant main effect of performance group (*F*(2,35) = 4.37, *p* = .020, ɳ^2^ = 0.20). This difference in FC between younger and older participants was specific to older poor performers, as revealed by a nonsignificant difference between younger and older good performers (*p* = .629). A nonsignificant performance group*thalamic subregion interaction (*F*(4.1, 72.47) = 1.657, *p* = .078, ɳ^2^ = 0.09) suggested that these differences were not strongly driven by any specific thalamic nuclei.

**Figure 6:**
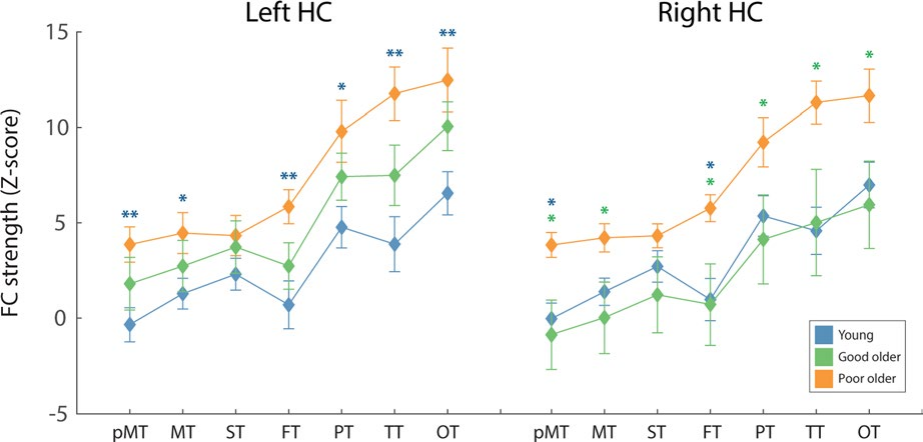
The average FC between each thalamic sub‐region and left and right hippocampus (HC) for younger participants (blue), good older PAL performers (green) and poor older PAL performers (orange). Asterisks depict a significant difference between (1) poor older PAL performers and younger participants (blue) and (2) poor older PAL performers and good older PAL performers (green). For left HC, poor older PAL performers exhibited significantly greater thalamic‐HC FC compared to younger participants (for all thalamic sub‐regions other than ST). For right HC, poor older PAL performers exhibited significantly greater thalamic‐HC FC compared to good older PAL performers (for all thalamic sub‐regions other than ST) and younger adults (pMT‐ and FT‐hippocampal FC). Error bars represent standard error, calculated across participants. *p < .05, **p < .01, ***p < .005 after FDR correction

Analysis of thalamo‐left hippocampal FC revealed similar patterns of thalamo‐hippocampal FC, as indicated by a main effect of performance group (*F*(2,35) = 5.26, *p* = .01, ɳ^2^ = 0.23).Older poor PAL performers exhibited greater FC compared to younger participants (*p* = .003) but not older good performers (*p* = .18). Again, the FC of older good performers and younger participants did not differ significantly (*p* = .137). A significant performance group*thalamic subregion interaction (*F*(4.41, 77.20) = 2.66, *p* = .034, ɳ^2^ = 0.13) suggested that this effect was driven by the thalamo‐hippocampal FC of the OT, FT, PT, Pre‐MT, and TT (see Figure 6b). Pairwise comparisons for each thalamic subregion, for left and right hippocampi, are shown in Table 2 and Figure 6.

**Table 2:** Thalamo‐hippocampal FC compared between old poor PAL performers and (1) younger participants and (2) old good PAL performers, for each thalamic subregion. Standard error is shown in brackets. **p < .01, *p < .05 (after FDR correction)

	Group a	Group b	Right HC	Left HC
			Mean difference (Group a–b)	Mean difference (Group a–b)
pMT	Old poor	Young	3.87 (1.43) *	4.20 (1.40) **
		Old good	4.70 (1.68) *	2.06 (1.65)
MT	Old poor	Young	2.83 (1.40)	3.18 (1.38) *
		Old good	4.19 (1.64) *	1.73 (1.62)
ST	Old poor	Young	1.61 (0.88)	2.03 (1.41)
		Old good	3.10 (1.77)	0.60 (1.65)
FT	Old poor	Young	4.79 (1.78) *	5.15 (1.68) **
		Old good	5.05 (2.09) *	3.12 (1.97)
PT	Old poor	Young	3.87 (1.97)	5.02 (1.78) *
		Old good	5.10 (2.31) *	2.37 (2.09)
TT	Old poor	Young	6.72(2.21) *	7.88(2.08) **
		Old good	6.28 (2.60) *	4.27 (2.44)
OT	Old poor	Young	4.67 (2.08)	5.92 (1.85) **
		Old good	5.71 (2.44) *	2.41 (2.17)

HC, hippocampus; pMT, premotor cortex; MT, primary motor cortex; ST, somatosensory cortex; FT, frontal cortex; PT, posterior parietal cortex; TT, temporal cortex; OT, occipital cortex.

#### 3.4.2 Thalamic‐motor FC and SRT performance

**Figure 7:**
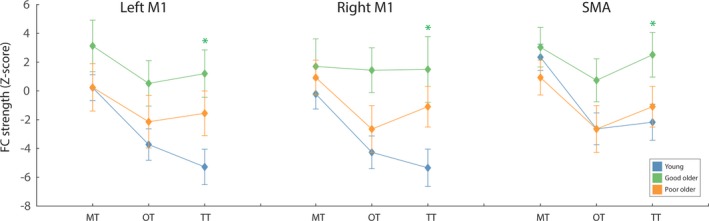
The average FC between each thalamic sub‐region and each motor RSN node, for young participants (blue), poor (slower) older SRT performers (orange) and good (faster) older SRT performers (green). Older good SRT performers exhibited significantly greater TT‐ motor cortex FC compared to young adults. Error bars represent standard error, calculated across participants. *p < .05, after FDR correction

A significant interaction between thalamic nuclei* performance group (*F*(2.85, 48.40) = 3.83, *p* = .017, ɳ^2^ = 0.18) revealed that, for older good SRT performers, FC was specifically greater between TT‐motor cortex (*p* = .018, ɳ^2^ = 0.21) compared with younger adults. The nonsignificant RSN node*thalamic nuclei*performance group (*F*(6.28, 106.71) = 1.29, *p* = .269, ɳ^2^ = 0.07) suggested that this difference between age‐groups was not specific to any motor RSN node. Pairwise comparisons confirmed that older fast performers had significantly greater TT‐left M1 (*p* = .003), TT‐right M1 (*p* = .011), and TT‐SMA (*p* = .020), compared to younger participants. Older poor SRT performers did not differ in FC compared to younger participants (*p* = .446, ɳ^2^ = 0.05) or older good SRT performers (*p* = .611, ɳ^2^ = 0.14). See Figure 7 for comparison of thalamic‐motor cortex FC between younger participants and good/poor performing older participants.

#### 3.4.3 Correlations between thalamic FC and behavioral performance

For PAL, both thalamo‐left and thalamo‐right hippocampi FC did not correlate significantly with the PAL performance measure (number of errors made on stage 6) in either younger or older adults.

For SRT, thalamo‐motor FC did not correlate significantly with SRT performance (average RT), for any of the three thalamic nuclei (MT, OT, and TT) in either younger or older adults. See Table 3 for the results from these correlational analyses.

**Table 3:** Outcomes from the partial correlational analyses between (a) thalamo‐hippocampal FC and memory performance and (b) thalamo‐motor cortex FC and simple reaction time (SRT) performance, for younger and older participants separately. For all correlations, age, gender, and estimated IQ (National Adult Reading Test score) were included as covariates of no interest. Values in the table are Pearson's *r* (depicted in italics), and the corresponding alpha level is reported below

		pMT	MT	ST	FT	PT	TT	OT
(a) Memory performance (number of errors on Paired Associates Learning task (PAL) at level 6) correlated with thalamo‐hippocampal FC, for each thalamic nuclei. For younger participants, the three participants with scores that would have categorized them as “older poor” performers were excluded in this analysis (see Methods)								
Left hippocampi								
Younger	*r*	*−.20*	*−.27*	*−.29*	*−.27*	*−.52*	*−.38*	*−.56*
	*p*	.47	.33	.29	.34	.05	.16	.04
Older	*r*	*.15*	*.12*	−*.07*	*.20*	*.06*	*.13*	*.09*
	*p*	.57	.64	.78	.44	.81	.63	.73
Right hippocampi								
Younger	*r*	*−.02*	*−.01*	*−.13*	*−.45*	*−.30*	*−.39*	*−.34*
	*p*	.99	.65	.65	.88	.28	.15	.22
Older	*r*	*.50*	*.37*	*.26*	*.41*	*.25*	*.22*	*.33*
	*p*	.04	.14	.32	.10	.31	.40	.20

		MT	OT	TT
(b) Average RT from the SRT task correlated with thalamo‐motor cortex FC, for each first‐order thalamic nuclei (MT, OT, TT) and for each motor cortex ROI				
Left M1				
Younger	*r*	*−.29*	*−.40*	*−.06*
	*p*	.31	.15	.83
Older	*r*	*−.43*	*−.19*	*−.26*
	*p*	.09	.46	.32
Right M1				
Younger	*r*	*−.38*	*−.55*	*−.55*
	*p*	.18	.04	.04
Older	*r*	*−.32*	*−.29*	*−.24*
	*p*	.22	.26	.35
SMA				
Younger	*r*	*−.05*	*.22*	*−.52*
	*p*	.87	.45	.86
Older	*r*	*−.21*	*−.24*	*−.27*
	*p*	.41	.35	.30

pMT, premotor cortex; MT, primary motor cortex; ST, somatosensory cortex; FT, frontal cortex; PT, posterior parietal cortex; TT, temporal cortex; OT, occipital cortex.

## 4 DISCUSSION

We investigated age‐related differences in thalamo‐cortical and thalamo‐hippocampal functional connectivity (FC) and their association with age‐related differences in cognitive performance on a memory and SRT task. Our results highlight that advanced age was associated with poorer performance on a visual–spatial memory task, as has been shown previously (Hayat et al., 2014; Lee, Archer, Wong, Chen, & Qiu, 2013; Rabbitt & Lowe, 2000). In addition, we provide evidence that, although visual–spatial memory performance was not directly correlated with thalamo‐hippocampal FC, older poor memory performers exhibited significantly greater thalamo‐right hippocampal FC compared to both younger adults and older good memory performers. Interestingly, this is in contrast to the results of comparing the two groups as a whole, which did not differ significantly in terms of thalamo‐right hippocampal FC strength. This suggests that thalamo‐hippocampal FC increases as a function of age, but that increases in thalamo‐right hippocampal FC may be more detrimental to memory performance compared to increases in thalamo‐left hippocampal FC. These results highlight the potential behavioral importance of thalamic FC, as well as demonstrating that increases in FC are not necessarily advantageous. Similar findings were reported by Ystad et al. (2010) who found that dorsomedial thalamus–striatum FC was negatively related to verbal episodic memory functioning in a sample of 49‐ to 80‐year‐olds. Although the hippocampus has long been implicated in memory processes (Riedel et al., 1999; Schacter et al., 1996; Squire, 1992), with a specific role in spatial memory and processing (Bird & Burgess, 2008; Eichenbaum et al., 1999; Henry, Petrides, St‐Laurent, & Sziklas, 2004), the role of the thalamus in memory processes should not be overlooked. Aggleton (2014) proposes that there are three parallel, yet distinct, “information streams” within the anterior thalamic nucleus (ATN) which integrate and work together to support episodic memory, while Nishio et al. (2014) demonstrated that the disruption of multiple thalamo‐cortical circuits can lead to prefrontal cortex dysfunction and memory deficits. This is supported by numerous studies which have highlighted the importance of thalamic‐PFC connectivity for memory and cognition (Cross, Brown, Aggleton, & Warburton, 2012; Funahashi, 2013; Gaffan, Murray, & Fabre‐Thorpe, 1993; Watanabe & Funahashi, 2012). Further research is required to fully understand how age may impact on these hippocampal‐thalamic‐PFC networks, and how their potential reorganization with age may impact on memory performance. Our results provide a starting point for this research, by indicating the feasibility and benefit of parcellating the thalamus in terms of identifying age‐related alterations to FC but also in distinguishing between good and poor memory performers. Future research should also establish whether age‐related differences in thalamo‐hippocampal FC are associated with other forms of memory or whether this finding is specific to visual–spatial memory. Similarly, future research should also investigate how thalamo‐hippocampal patterns may differ between thalamic subregions, as a function of differential memory processes.

In addition to poorer visual–spatial memory performance with age, we observed slowing of information processing in older adults, as assessed by the SRT task, and demonstrated that older faster SRT performers exhibited significantly greater thalamic‐motor cortex FC compared to younger adults, suggesting that faster SRT performance was associated with greater thalamic‐motor cortex FC. Although the FC in older fast performers was not significantly different to older slow performers, older slow performers did not exhibit significantly greater FC compared to younger participants, which suggests that greater thalamus–motor cortex FC may be more strongly related to SRT performance than age. This lack of differentiation between the two older groups could be due to statistical power; as by splitting our older group into good and poor performers, we reduced the group sizes to 10 participants instead of 20. Future research should replicate these results in larger groups of older participants, in order to further establish whether greater thalamo‐motor FC in older age is specific to older fast performers. Alternatively, a full explanation of these differences may require a more holistic investigation of the motor system, of which the thalamo‐cortical interactions we have examined are only a part.

The role of thalamic‐motor RSN connectivity on SRT performance and the potential modulation with age is a finding that warrants further investigation. To date, functional links between the thalamus and motor cortex have been identified using DTI and fMRI (Guye et al., 2003; Hale et al., 2015; Lehéricy et al., 2006; Zhang et al., 2008) and anatomical evidence has shown that the thalamus is substantially connected to subcortical motor regions (Sherman & Guillery, 2013; pg. 169). Despite this known thalamic‐motor connectivity, few studies have investigated their role in measures of reaction time (RT). Those that have had identified associations between RT and thalamic white matter connectivity, diffusivity, and gamma oscillations (Brucke et al., 2013; Fall, Querne, Le Moing, & Berquin, 2015; Tuch et al., 2005). Taken together, this evidence suggests that the thalamus is well situated to contribute to individual differences in RT as well as age‐related slowing, via reorganization of thalamic connectivity or structural and/or functional thalamic changes with age. Future research should look to probe thalamic‐motor cortex connectivity more specifically using segmentation of the thalamus to investigate the connectivity between individual thalamic subregions and motor cortex.

In older adults, increased activity (Buckner, 2004; Cabeza, Anderson, Locantore, & McIntosh, 2002; Reuter‐Lorenz et al., 2000) or connectivity (Campbell, Grady, Ng, & Hasher, 2012; Davis, Dennis, Daselaar, Fleck, & Cabeza, 2008; Geerligs, Maurits, Renken, & Lorist, 2014) is often considered to be compensatory in nature. The recruitment of additional brain regions or increased connectivity between brain regions has been suggested to support the maintenance of cognitive function which would otherwise be disrupted due to age‐related brain changes, such as loss of gray matter or reductions in within‐network connectivity. However, other evidence has shown that increased FC does not always equate to better performance (Geerligs, Saliasi, Maurits, Renken, & Lorist, 2014; Grady et al., 2010; Salami, Eriksson, & Nyberg, 2012). One explanation of this finding could be that older age is associated with reduced specificity of brain networks, which results in less efficient processing, potentially by increasing interference between network activity (Baltes & Lindenberger, 1997) and thus causing deficits in cognition (Antonenko & Floel, 2014). Our results provide evidence for both scenarios and suggest that the relationship between changes in brain networks and behavioral performance with age may be quite specific to individual behavioral domains. We found that faster RT in older adults was associated with greater thalamic‐motor cortex FC, compared to younger adults, while greater thalamo‐hippocampal FC (particularly from the thalamic subregion that connects mainly to the temporal lobe) in older adults was associated with poorer memory performance. Further research is required to investigate the differential effects of increased or decreased thalamo‐cortical connectivity with age on cognition, their domain specificity, as well as the relationship between changes in task‐related activations and changes in FC.

In this study, we identified differences in absolute thalamic FC strength between younger participants, older good, and older poor performers but did not identify significant correlations between thalamic FC strength and behavioral performance when the two measures were on a continuous scale. This discrepancy may be due to a number of methodological factors, such as reduced variability in either FC, behavioral performance, or both, due to our sample size, which meant that the spread of performance vs. FC was not large enough to produce a correlation that was significant. Alternatively, the relationship between thalamic FC and behavioral performance may not be linear across age, or even directly related. For example, greater thalamo‐hippocampal FC in older adults may interfere with memory performance in a more nuanced manner, by affecting some unknown process(es), required for memory, rather than FC strength being directly proportional to the number of items forgotten. Replication of this study, with larger sample sizes and a wide range of younger and older adults combined with a broader range of cognitive tasks, may help us understand more clearly the relationship between thalamic FC and behavioral performance and how that relationship may change with age. One interesting observation that warrants further investigation is the fact that, although there was some overlap, the performance groups for the two tasks were not homogenous. Only five of the eleven “poor” memory performers were also in the “slow” SRT group, while four of the nine “good” memory performers were in the “fast” RT group. Investigating differential cognitive performance and associations with FC within an individual may help us understand whether age‐related decreases in network segregation affects networks differently between individuals or whether the network disruption is similar across individuals but the effect on cognition is heterogenous.

By performing FC analysis using thalamic subregions, we were able to present more specific results of the effect of age on thalamic FC, compared to results using thalamic masks which treat the thalamus as a homogeneous structure, which, in our study, showed a less clear effect of age (See Appendix S1). Many studies have now provided evidence that it is possible to segment the thalamus using noninvasive DTI and fMRI data into subregions which largely correspond with known subdivisions identified from anatomical and histological evidence (Hale et al., 2015; Jang & Yeo, 2014; Kumar et al., 2014; Zhang, Snyder, Shimony, Fox, & Raichle, 2010; Zhang et al., 2008). However, Hale et al. (2015) highlight the differences between analysis methods even within a single imaging modality. Although their results suggest that ICA may provide a more specific definition of thalamic subregions, we chose to use a structural atlas to segment the thalamus for the following reasons: (1) It is less intuitive to interpret ICA results for defining thalamic subregions, particularly when comparing between age‐groups; (2) Hale et al. reported there was largely a correspondence between the results from the structural and ICA definitions, suggesting that the added specificity provided by ICA may not warrant the additional interpretation complexity for this preliminary study.

One potential limitation of the current study is the presence of non‐neuronal confounds in fMRI connectivity measurements, which may artificially induce, or exaggerate, differences between age‐groups, as highlighted by Balsters et al. (2013). In order to account for differences in breathing and heart rate across age‐groups, and individuals, we collected both respiratory and cardiac pulse data for all participants and regressed these from participant's functional data. Nonetheless, the possibility of age‐related differences in other non‐neuronal factors, such as vasculature and cerebral blood flow (CBF) (Beason‐Held et al., 2012; Peters, 2006; Riddle, Sonntag, & Lichtenwalner, 2003), may have had an impact. However, a recent study revealed that, although older adults did exhibit reduced CBF in comparison with younger adults, the uptake of oxygen, lactate, and glucose did not differ between the two age‐groups, suggesting that reduced CBF in older adults does not affect the brain's ability to uptake nutrients (Fisher et al., 2013). Although, others have reported that regionally specific age differences in physiological fluctuations exist, which may only partly reflect those captured on a global level (Tsvetanov et al., 2015). This means that global regression methods may not always be the most accurate method of physiological correction between age‐groups as any regional differences that deviate from the global pattern may be inadequately corrected for. However, for this data set, resting‐state fluctuation amplitude (an index of vascular reactivity) did not show any regional age differences (data not presented here). Nonetheless, novel modeling methods such as generative modeling, which allows for the effects of neural connectivity to be separated from the hemodynamic component, have previously been demonstrated to be useful in studies of older adults (Tsvetanov et al., 2016) and should be considered as an alternative to the global removal method we utilized in this study. These factors remain complex methodological issues for the field of brain aging research that require further investigation. The use of EEG‐fMRI or arterial spin labeling, which provides a more direct and quantifiable measure of cerebral hemodynamics, may also go some way to addressing such potential differences between age‐groups.

An additional caveat of investigating differences in brain function with advancing age is differences in gray‐matter volumes between age‐groups and the variability of such age‐related differences between individuals. Studies using gray‐matter volume as voxel‐based regressors have provided evidence that some functional differences between age‐groups can be a consequence of gray‐matter atrophy (Kalpouzos, Persson, & Nyberg, 2012), while others persist after correction for gray‐matter volume (Salami et al., 2012). In this study, we addressed differences in gray‐matter volumes within cortical ROIs (and the hippocampus) using partial volume maps following segmentation to exclude any voxels not classified as gray‐matter from any analyses. However, segmentation of subcortical structures, such as the thalamus, can be less reliable and gray‐matter is often misclassified as white. For this reason, we chose to exclude any CSF voxels, to go some way to addressing differences in thalamic volume between the two age‐groups, but, currently, this remains a methodological issue for researchers investigating thalamic connectivity differences in older age.

Detailed electrophysiological and histological work (Steriade & Deschenes, 1984), combined with more recent studies linking neuroimaging with behavioral measures, has vastly increased our understanding of thalamo‐cortical connectivity over the past few decades. It is now apparent that the thalamus plays an important role not only in integrating and transmitting sensory information, but also in regulating cortical regions and both directly and indirectly supporting cortico‐cortical connectivity. Understanding the connectivity between brain regions is imperative to understanding brain function. A systematic review of the functional neuroanatomy of the thalamus by Power and Looi (2015) highlighted that, although the precise role of the thalamus remains unclear, its importance in the functional connectome is beyond doubt. Some have argued that a significant factor in determining the functions that any cortical region is capable of is its connectivity to the thalamus (Sherman & Guillery, 2013). However, there are still a number of questions to be answered regarding (1) how these connections support cognitive function, (2) how changes with age or disease disrupt thalamic connectivity to both cortical and subcortical brain regions, and (3) the impact of such connectivity changes on cognition.

Our work has provided new evidence of the potential role of thalamo‐cortical and thalamo‐hippocampal connectivity in supporting reaction times and memory in aging. As evidence mounts, it seems unlikely that a single thalamic nucleus is responsible for a specific cognitive ability or memory function, and a distributed system appears more probable where the integration of information and connectivity across thalamic, subcortical, and cortical regions is involved in a range of cognitive abilities (Mitchell & Chakraborty, 2013; Mitchell & Dalrymple‐Alford, 2006). The role of the thalamus in terms of aging, disease, and cognition should not be underestimated, and future research should look to integrate measures of the thalamus alongside cortical networks which are often the focus of studies of cognition.

## ACKNOWLEDGMENTS

This work was supported by the UK Engineering and Physical Sciences Research Council (EPSRC) (grant number EP/J002909/1); EPSRC (grant number EP/I022325/1) to S.D.M. and Birmingham University Fellowship to S.D.M. AG was funded by an Economic and Social Research Council PhD studentship (ES/J50001X/1). Participants did not consent to their data being made openly available. Further information about the data and conditions for access are available at the University of Birmingham Research at Birmingham website at http://rab.bham.ac.uk/.

## AUTHOR DISCLOSURE STATEMENT

No competing financial interests exist.

## APPENDIX 1

### 1.1

**Table A1:** MNI coordinates of the peak voxel for each RSN node, around which 5 × 5 × 5 voxel ROIs, were created. Final ROI size (group mean number of voxels and standard deviation across participants) after transforming ROIs into individual space and selecting only gray‐matter voxels is displayed

	*x*	*y*	*z*	Average ROI size ± *SE*	
				Younger	Older
Auditory					
Left STG	75	54	39	71 ± 8.32	58 ± 5.73
Right STG	17	53	39	72 ± 5.99	61 ± 9.22
Motor					
Left M1	67	55	67	54 ± 5.53	43 ± 6.33
Right M1	23	55	67	46 ± 7.32	38 ± 5.82
SMA	45	53	65	61 ± 9.25	50 ± 9.55
Visual					
Left lateral	69	26	41	67 ± 7.34	59 ± 8.35
Right lateral	21	29	41	71 ± 6.51	62 ± 8.13
Left primary	49	27	39	54 ± 8.23	48 ± 7.51
Right primary	39	29	39	51 ± 5.68	43 ± 6.19

**Table A2:** Final ROI size (group mean number of voxels and standard deviation across participants) after transforming anatomically defined ROIs into individual space and selecting only gray‐matter voxels (or excluding CSF voxels for thalamic regions) is displayed

	Average ROI size ± *SE*	
	Younger	Older
Hippocampus		
Left HC	90.35 ± 6.85	73 ± 9.46
Right HC	91.15 ± 5.78	75 ± 7.93
Thalamic subregions		
OT	162 ± 15.52	137 ± 16.84
FT	575 ± 55.20	514 ± 61.27
PT	403 ± 14.45	346 ± 38.88
Pre‐MT	156 ± 24.96	149 ± 11.08
MT	269 ± 15.52	260 ± 17.93
ST	154 ± 15.25	140 ± 11.22
TT	553 ± 60.08	456 ± 81.01
