# Supplementary material for: Thalamic functional connectivity and its association with behavioral performance in older age
Source: Brain Behav. 2018 Feb 27;8(4):e00943. doi: 10.1002/brb3.943 (PMC5893345; doi:10.1002/brb3.943)
Supplement: Supplementary file 1 [file BRB3-8-e00943-s001.docx]

**Additional analyses**

*Thalamic FC*

We present the results seeding from an anatomically defined thalamic mask (i.e. where the thalamus is treated as one homogeneous region rather than segmented into sub-regions).

Thalamus – sensory RSNs

The average FC between the thalamus and each sensory RSN is presented in S3. A mixed-design ANOVA revealed no significant difference in average FC across sensory RSNs (auditory, motor, visual) between the two age groups as indicated by a non-significant main effect of age group (F(1,38)=2.69, p=0.109, *ɳ^2^*=0.066) and a non-significant age*network interaction (F(1.67, 63.29)=0.13, p=0.841, *ɳ^2^*=0.003). Figure S4 depicts the FC between the thalamus and each sensory RSN node, which are averaged to create the composite thalamic FC scores presented in S5.

Thalamo-hippocampal FC

Average thalamo-hippocampal FC is presented in S5 for the combined bilateral hippocampi and for left and right hippocampi individually. Older adults were found to exhibit greater thalamo-hippocampal FC (averaged across left and right hippocampal nodes), compared to younger adults (F(1,38)=6.63,p=0.014).


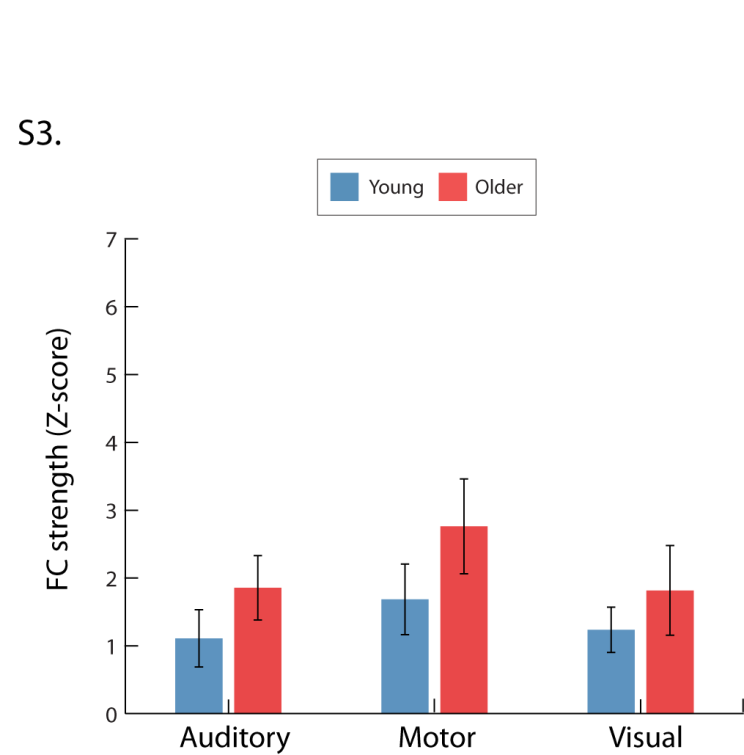


S3: The average thalamus-sensory RSN FC for the two age groups. For each RSN, FC was first averaged across left and right thalamic nodes, and then across each individual RSN node, to create an average RSN FC strength. Error bars represent standard error, calculated across participants.


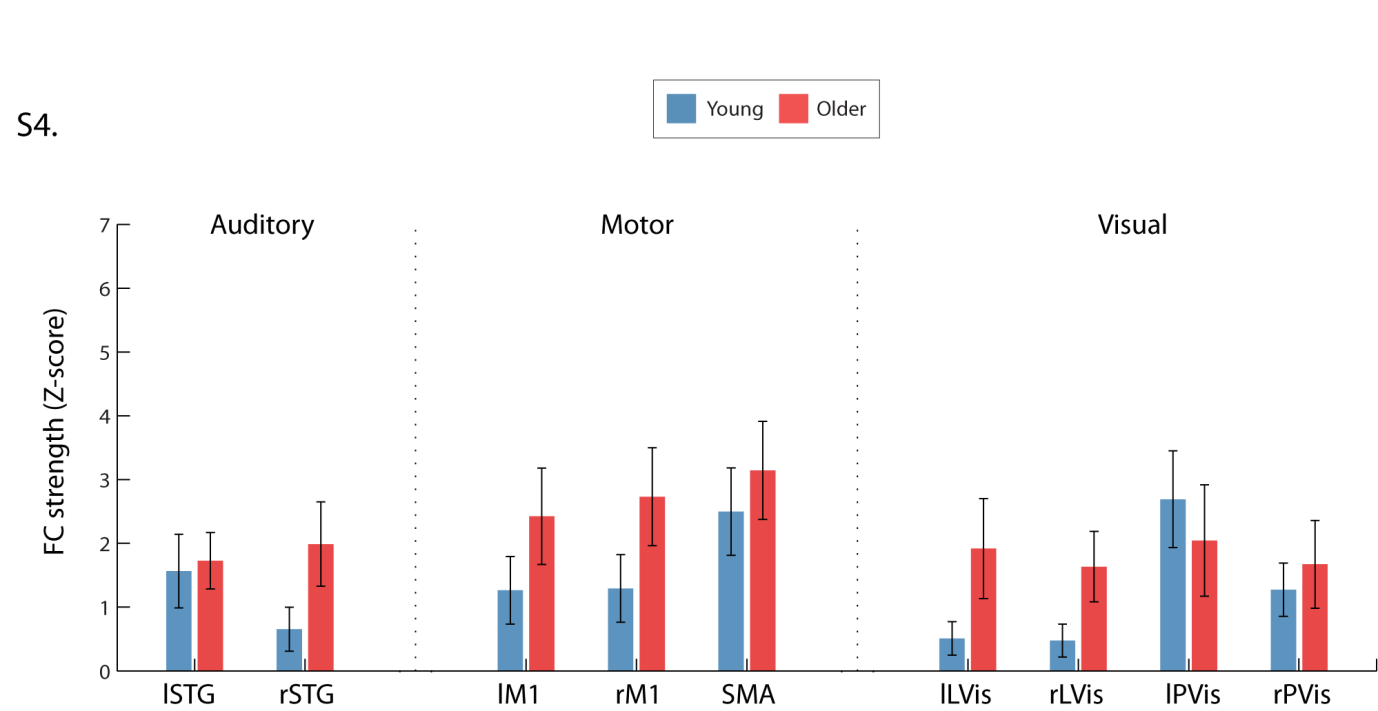


S4: The average FC between thalamus (averaged across left and right nodes) and the individual nodes of the auditory, motor and visual RSNs. Average FC across the individual nodes of each RSN form the average thalamus-RSN FC depicted in S5. Error bars represent standard error, calculated across participants.


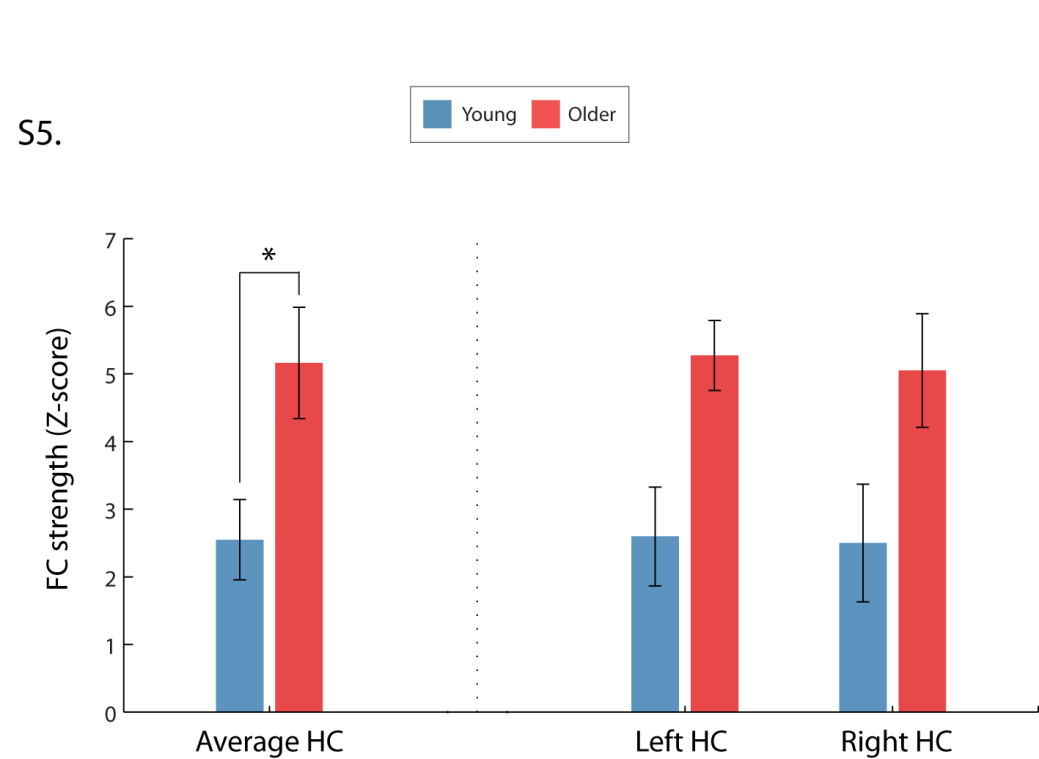


S5: The average thalamus-hippocampal FC for the whole hippocampus, as well as left and right separately, for the two age groups. *p<0.05. Error bars represent standard error, calculated across participants.
